# Supplementary figures and images for: miR-410-3p is induced by vemurafenib via ER stress and contributes to resistance to BRAF inhibitor in melanoma
Source: PLoS One. 2020 Jun 17;15(6):e0234707. doi: 10.1371/journal.pone.0234707 (PMC7299409; doi:10.1371/journal.pone.0234707)

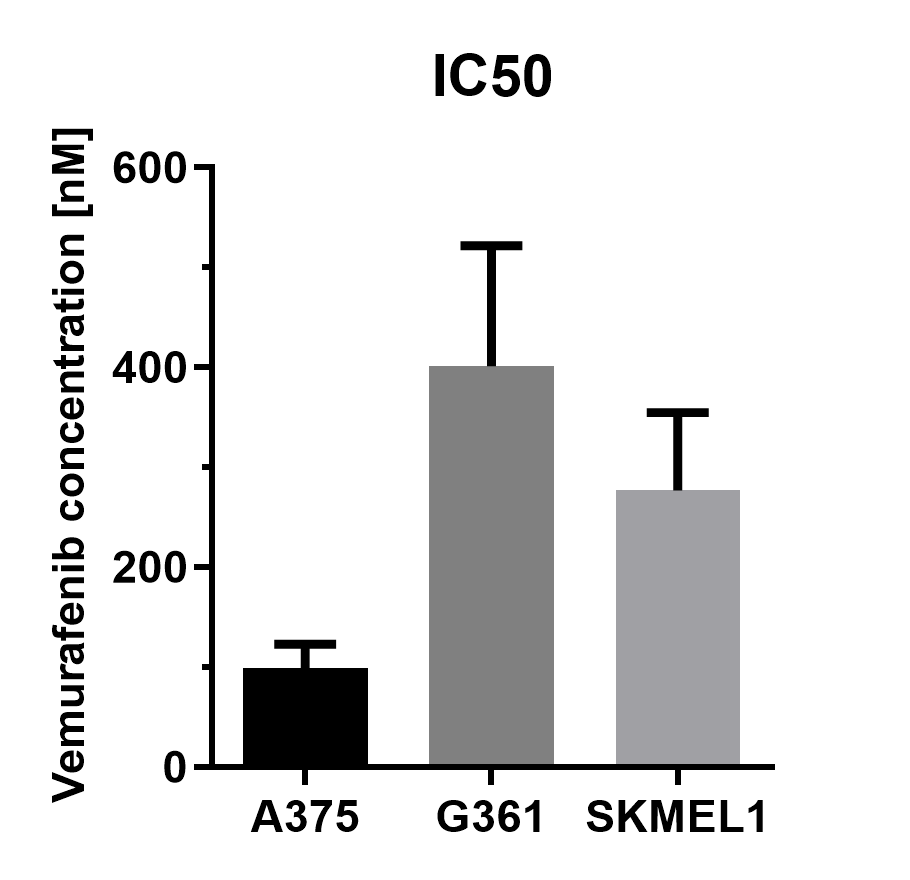

Supplement: S1 Fig — (TIFF) [file pone.0234707.s001.tiff]

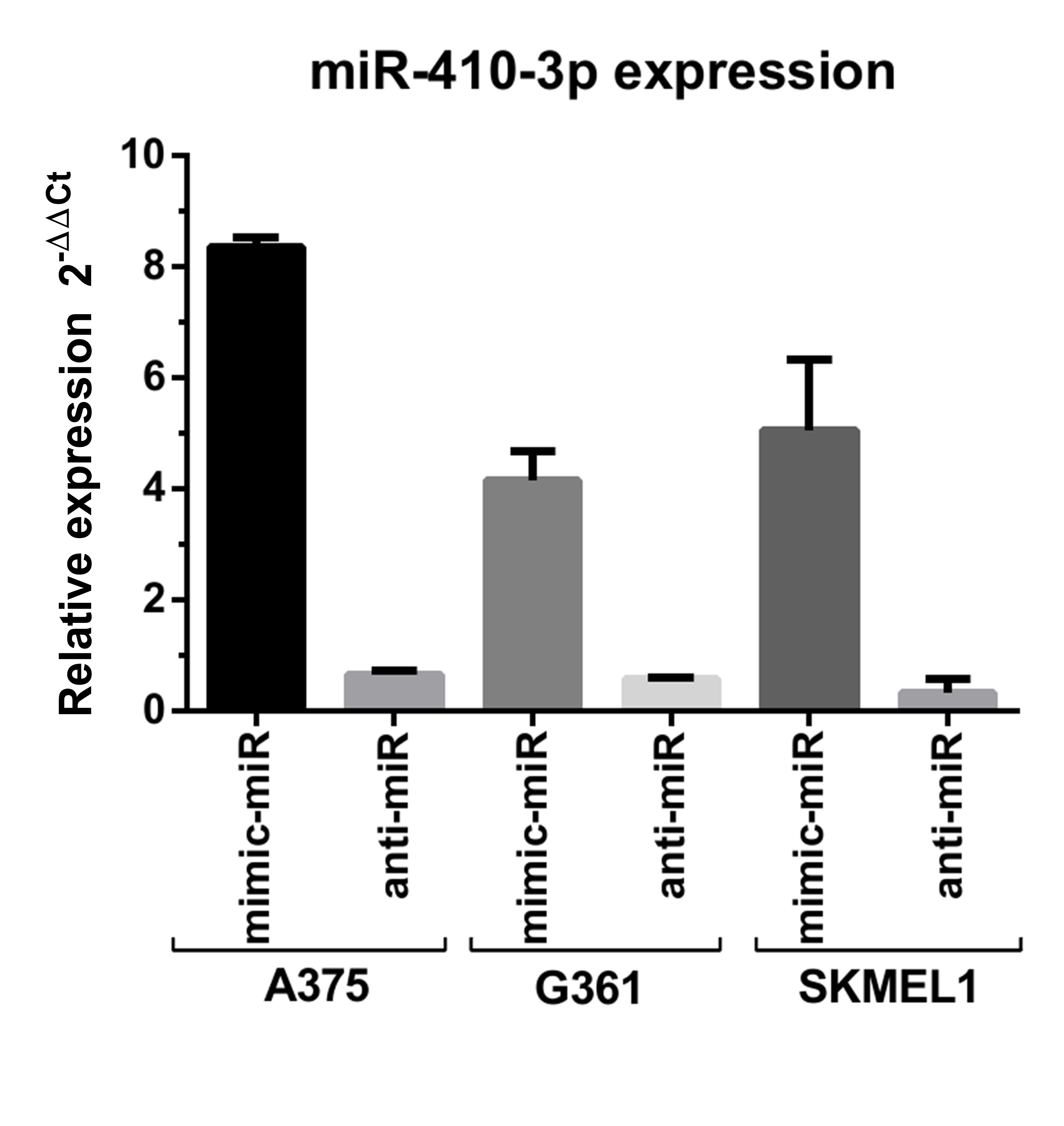

Supplement: S2 Fig — The efficiency of the transfection was determined using qPCR. The expression of miR-410-3p is presented as relative expression compared to the miR-scrambled for mimic-miR-410-3p and anti-miR-scrambled for anti-miR-410-3p. (TIFF) [file pone.0234707.s002.tiff]

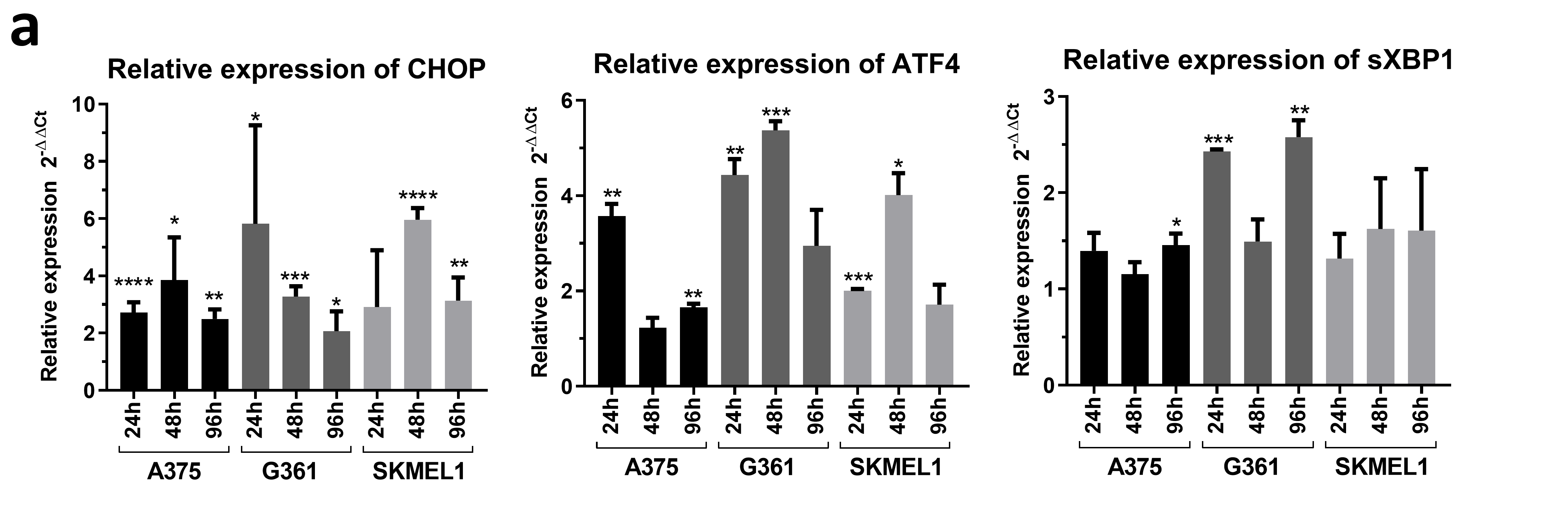

Supplement: S3 Fig — The expression of ER stress markers are presented as relative expression compared to vehicle (DMSO)-treated cells. *—p<0.05, **—p<0.01, ***—p<0.001, ****—p<0.0001. (TIFF) [file pone.0234707.s003.tiff]

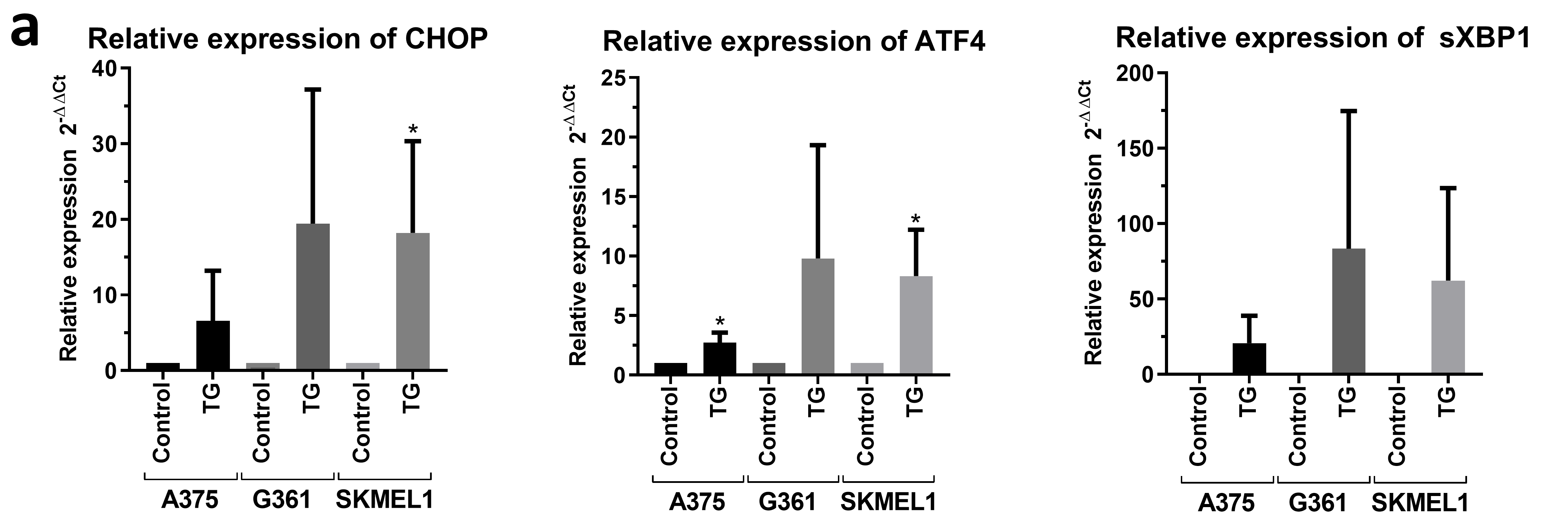

Supplement: S4 Fig — The expression of ER stress markers, CHOP, ATF4 and sXBP1 was determined using qPCR. *—p<0.05. (TIFF) [file pone.0234707.s004.tiff]
