# Supplementary material for: miR-410-3p is induced by vemurafenib via ER stress and contributes to resistance to BRAF inhibitor in melanoma
Source: PLoS One. 2020 Jun 17;15(6):e0234707. doi: 10.1371/journal.pone.0234707 (PMC7299409; doi:10.1371/journal.pone.0234707)
Supplement: S1 Table — (DOCX) [file pone.0234707.s005.docx]

**Supplementary table 1. Clinical data of the patients involved in the study.**

| **Patient** | **Mel001** | **Mel002** | **Mel003** | **Mel004** | **Mel005a** | **Mel005b** | **Mel006** | **Mel007** | **Mel008** | **Mel009** | **Mel010** | **Mel011** | **Mel012** |
| --- | --- | --- | --- | --- | --- | --- | --- | --- | --- | --- | --- | --- | --- |
| Sex | F | M | F | M | F | F | F | F | M | F | F | F | M |
| Age | 78 | 81 | 59 | 66 | 85 | 85 | 88 | 65 | 76 | 84 | 82 | 76 | 88 |
| Anatomical location | Left cheek | Right cheek | n.d. | Back | Left elbow | Left arm | Foot | Abdomen | Left temporoparietal region | Cheek | Left eyebrow | Left crus | Chest |
| Histological subtype | Fusocellular NM | Fusocellular NM | Epithelioid-fusocellular NM | Fusocellular NM | NM | LMM | Fusocellular NM | Amelanotic NM | NM | Fusocellular NM | SSM | LMM | NM |
| TNM | pT4b | pT4b | pT3b | pT4bN0 | pT4bN0 | pT1aN0 | pT4bN2b | pT3b | pT3b | pT3a | pT1b | pT1a | pT4b |
| Clark | V | V | III | IV | n.d. |  | IV | IV | IV | IV | II | II | IV |
| Breslow | 8 mm | 7 mm | 3.5 mm | 4.5 mm | 18 mm | <1 mm | 8 mm | 3 mm | 22 mm | 2.5 mm | 0.9 mm | 0.29 mm | 9 mm |
| Ulceration | Yes | Yes | Yes | Yes | Yes | No | Yes | Yes | Yes | No | Yes | No | Yes |
| Mitotic index | 3-6/mm^2^ | 16 MF / 10 HPF | 29 MF / 10 HPF  5/mm^2^ | 22 MF / 10 HPF 7/mm^2^ | 7 MF/ 10 HPF | n.d. | 40 MF / 10 HPF | 4/mm^2^ | 1/mm^2^ | 6/mm^2^ | 2/mm^2^ | 1/mm^2^ | 25/mm^2^ |
| Lymphoid infiltration | Yes | Yes | Yes | Yes | Yes | n.d. | Yes | n.d. | No | Non-brisk | Brisk | Brisk | n.d. |
| Satellite tumors | In subcutaneous fat tissue | No | No | No | No | n.d. | No | No | No | No | No | No | No |
| Lymph nodes | n.d. | Clear | n.d. | Clear | Clear | n.d. | Metastases in 2/9 | n.d. | n.d. | n.d. | n.d. | n.d. | n.d. |

**Abbreviations:** n.d. – not defined, MF – mitotic figures, HPF – high power fields, NM – nodular melanoma, LMM – lentigo maligna melanoma, SMM – superficial spreading melanoma
