# Supplementary material for: miR-410-3p is induced by vemurafenib via ER stress and contributes to resistance to BRAF inhibitor in melanoma
Source: PLoS One. 2020 Jun 17;15(6):e0234707. doi: 10.1371/journal.pone.0234707 (PMC7299409; doi:10.1371/journal.pone.0234707)
Supplement: S2 Table — (DOCX) [file pone.0234707.s006.docx]

**Supplementary Table 2. Results from the enrichment analysis of miR-410-3p targets in KEGG pathways using starBase.**

| Pathway name | log10 (p value) | log10 (FDR) | Gene number | Pathway gene number | Target gene number | Common gene number |
| --- | --- | --- | --- | --- | --- | --- |
| KEGG_Pathways_In_Cancer | -13,02073 | -10,75356 | 20628 | 328 | 4266 | 126 |
| KEGG_P53_Signaling_Pathway | -7,53416 | -5,56802 | 20628 | 69 | 4266 | 35 |
| KEGG_Neurotrophin_Signaling_Pathway | -6,93837 | -5,14832 | 20628 | 126 | 4266 | 52 |
| KEGG_Renal_Cell_Carcinoma | -6,18851 | -4,5234 | 20628 | 70 | 4266 | 33 |
| KEGG_Pancreatic_Cancer | -5,65415 | -4,29007 | 20628 | 70 | 4266 | 32 |
| KEGG_Cell_Cycle | -5,84126 | -4,27306 | 20628 | 128 | 4266 | 50 |
| KEGG_Chronic_Myeloid_Leukemia | -5,67647 | -4,25439 | 20628 | 73 | 4266 | 33 |
| KEGG_Prostate_Cancer | -5,67968 | -4,19066 | 20628 | 89 | 4266 | 38 |
| KEGG_Melanoma | -4,98987 | -3,67694 | 20628 | 71 | 4266 | 31 |
| KEGG_Adherens_Junction | -4,87765 | -3,61047 | 20628 | 75 | 4266 | 32 |
| KEGG_Endocytosis | -4,37583 | -3,28475 | 20628 | 183 | 4266 | 61 |
| KEGG_Colorectal_Cancer | -4,4018 | -3,28075 | 20628 | 62 | 4266 | 27 |
| KEGG_Ubiquitin_Mediated_Proteolysis | -4,41941 | -3,26618 | 20628 | 138 | 4266 | 49 |
| KEGG_Glioma | -4,43715 | -3,24916 | 20628 | 65 | 4266 | 28 |
| KEGG_Focal_Adhesion | -4,44794 | -3,22216 | 20628 | 201 | 4266 | 66 |
| KEGG_Small_Cell_Lung_Cancer | -4,13461 | -3,07156 | 20628 | 84 | 4266 | 33 |
| KEGG_Spliceosome | -3,9949 | -2,95817 | 20628 | 128 | 4266 | 45 |
| KEGG_Mtor_Signaling_Pathway | -3,96338 | -2,95148 | 20628 | 52 | 4266 | 23 |
| KEGG_Wnt_Signaling_Pathway | -3,92722 | -2,9388 | 20628 | 151 | 4266 | 51 |
| KEGG_Mapk_Signaling_Pathway | -3,70351 | -2,73736 | 20628 | 267 | 4266 | 80 |
| KEGG_Apoptosis | -3,67996 | -2,73501 | 20628 | 88 | 4266 | 33 |
| KEGG_Protein_Export | -3,51543 | -2,64619 | 20628 | 24 | 4266 | 13 |
| KEGG_Tgf_Beta_Signaling_Pathway | -3,51582 | -2,62886 | 20628 | 86 | 4266 | 32 |
| KEGG_Basal_Cell_Carcinoma | -3,52135 | -2,6159 | 20628 | 55 | 4266 | 23 |
| KEGG_Insulin_Signaling_Pathway | -3,53929 | -2,61454 | 20628 | 137 | 4266 | 46 |
| KEGG_Ascorbate_And_Aldarate_Metabolism | -3,2875 | -2,4353 | 20628 | 25 | 4266 | 13 |
| KEGG_Non_Small_Cell_Lung_Cancer | -3,20539 | -2,36958 | 20628 | 54 | 4266 | 22 |
| KEGG_Regulation_Of_Actin_Cytoskeleton | -2,95373 | -2,13372 | 20628 | 216 | 4266 | 64 |
| KEGG_Adipocytokine_Signaling_Pathway | -2,89535 | -2,09058 | 20628 | 67 | 4266 | 25 |
| KEGG_Melanogenesis | -2,7146 | -1,93879 | 20628 | 102 | 4266 | 34 |
| KEGG_Erbb_Signaling_Pathway | -2,71648 | -1,92643 | 20628 | 87 | 4266 | 30 |
| KEGG_Axon_Guidance | -2,43501 | -1,67299 | 20628 | 129 | 4266 | 40 |
| KEGG_Phosphatidylinositol_Signaling_System | -2,37682 | -1,62817 | 20628 | 76 | 4266 | 26 |
| KEGG_Valine_Leucine_And_Isoleucine_Degradation | -2,31686 | -1,58117 | 20628 | 44 | 4266 | 17 |
| KEGG_Cysteine_And_Methionine_Metabolism | -2,27941 | -1,55631 | 20628 | 34 | 4266 | 14 |
| KEGG_Rna_Degradation | -2,24261 | -1,53174 | 20628 | 59 | 4266 | 21 |
| KEGG_Endometrial_Cancer | -2,21709 | -1,51812 | 20628 | 52 | 4266 | 19 |
| KEGG_Pentose_And_Glucuronate_Interconversions | -2,18454 | -1,49715 | 20628 | 28 | 4266 | 12 |
| KEGG_Bladder_Cancer | -2,14213 | -1,47702 | 20628 | 42 | 4266 | 16 |
| KEGG_B_Cell_Receptor_Signaling_Pathway | -2,14404 | -1,46793 | 20628 | 75 | 4266 | 25 |
| KEGG_Inositol_Phosphate_Metabolism | -2,01921 | -1,36482 | 20628 | 54 | 4266 | 19 |
| KEGG_T_Cell_Receptor_Signaling_Pathway | -2,00517 | -1,36124 | 20628 | 108 | 4266 | 33 |
| KEGG_Type_Ii_Diabetes_Mellitus | -1,98877 | -1,35507 | 20628 | 47 | 4266 | 17 |
